# Stapleless Laparoscopic Sleeve Gastrectomy: Reasoning and Technical Insights

**DOI:** 10.1007/s11695-017-3058-y

**Published:** 2017-12-21

**Authors:** Matteo Catanzano, Lisa Grundy, Mohamed Bekheit

**Affiliations:** 1 Department of Surgery, Aberdeen Royal Infirmary, Foresterhill Health Campus, Aberdeen, AB252ZN UK; 2 Department of Surgery, El kabbary General Hospital, Alexandria, Egypt; 3 Institute of Medical Sciences, University of Aberdeen, Aberdeen, UK; 4 Centre Hépato-Biliaire, 12 av. Paul Vaillant Couturier, AP-HP, Hôpital Paul Brousse, 94800 Villejuif, France; 5 Inserm Unité 1193, 12 av. Paul Vaillant Couturier, 94800 Villejuif, France; 6 Ecole doctorale Innovation Therapeutique, Universite Pais-Sud, Châtenay-Malabry Cedex, France

**Keywords:** Stapleless, Staplerless, Stapler, Laparoscopic, Sleeve, Obesity, Bariatric

## Abstract

**Background:**

Laparoscopic sleeve gastrectomy (LSG) with staple line reinforcement (SLR) is a popular and safe treatment option for morbid obesity. We have developed, devised, and described our own method of stapleless laparoscopic sleeve gastrectomy, which in our limited study appeared safe, efficacious, and potentially cost-effective.

**Methods:**

We analyzed the outcome of our modified LSG in a case series of three middle-aged women (median age 42 years old). Our main modification was sutured closure of the stomach rather than the commonly utilized technique of stapled closure. Our primary measure of success was the occurrence of post-operative leak. Secondary measures were (a) length of operation, (b) duration of inpatient stay, and (c) percentage of weight loss at 6 and 12 months post operation.

**Results:**

Median operative time = 132 min (120–195 min), and median inpatient stays were 2 days. No post-operative leaks were recorded. The median excess weight loss at 6 months was 39% of initial weight loss and 57.7% at 12 months.

**Conclusions:**

Stapleless LSG has the potential to be an affordable alternative to the traditional LSG. High-powered studies and a formal cost analysis are required.

## Introduction

Laparoscopic sleeve gastrectomy (LSG) has become a popular choice for the treatment of morbid obesity and its related complications [1]. However, high rates of complications are related to the reliance on staple closure. Hemorrhage is an important and life-threatening complication with an incidence of 0–8.7%; the second prevalent complication is gastric leak with an incidence of 0–8% [2–5]. Subsequently, staple line reinforcement (SLR) techniques have emerged. These techniques aim to reduce the incidence of the aforementioned complications and include improving cartridge technology, oversewing staple lines, and utilizing staple line buttressing material, e.g., specific bioabsorbable material, such as glycolide-trimethylene carbonate copolymer (Gore Seamguard), bovine pericardium strips (Peristrips Dry and PSD Veritas), or porcine small intestinal submucosa (Surgisis Biodesign).

Given rising financial pressures on healthcare systems worldwide, together with rising rates of morbid obesity and associated co-morbidities, alternative and safe ways to carry out LSG are required. Currently, the most cost-effective reinforcement method is oversewing of the staple line.

In this proof of concept study, we analyzed the stapleless technique for laparoscopic sleeve gastrectomy where a pouch is created via energy-based resection, and the stomach is closed with sutures alone. Based on our previous experience [6], we hypothesize that stapleless sleeve gastrectomy could be safe and effective. Three patients underwent the procedure; we discuss logical critiques of our proposed modification.

## Materials and Methods

We started performing LSG in 2009, at the Department of Surgery, El Kabbary General Hospital, Alexandria, Egypt. After identifying high rates of leakage in our cohorts, we considered technical modifications to minimize complications. We previously reviewed the leakage rate after different bariatric procedures, and identified post-operative leaks were highest in patients post LSG [7]. This led to a series of modifications that resulted in a significant reduction in the leakage rate [8]. Analysis of these procedures identified invagination of sutures as a successful way of reinforcing LSG. This prompted us to question if staples were a necessary part of LSG. Acknowledging the expense of stapled closure, we postulated that forgoing staples could be a cost-effective and safe modification to LSG.

Hence, we devised a stapleless technique (described below). The procedure was carried out as a proof of concept on three middle-aged women (median age 42 years old), with the occurrence of post-operative leak as our primary outcome. The secondary outcomes were the operative time and the frequency of prolonged hospital stay. Informed consent was taken from all patients included in this study. Ethical approval was granted by the Ethical Committee of Human Research (IRB), Faculty of Medicine, Alexandria University, and in El Kabbary Hospital, Alexandria, Egypt.

### Description of the Technique

**Fig. 1:**
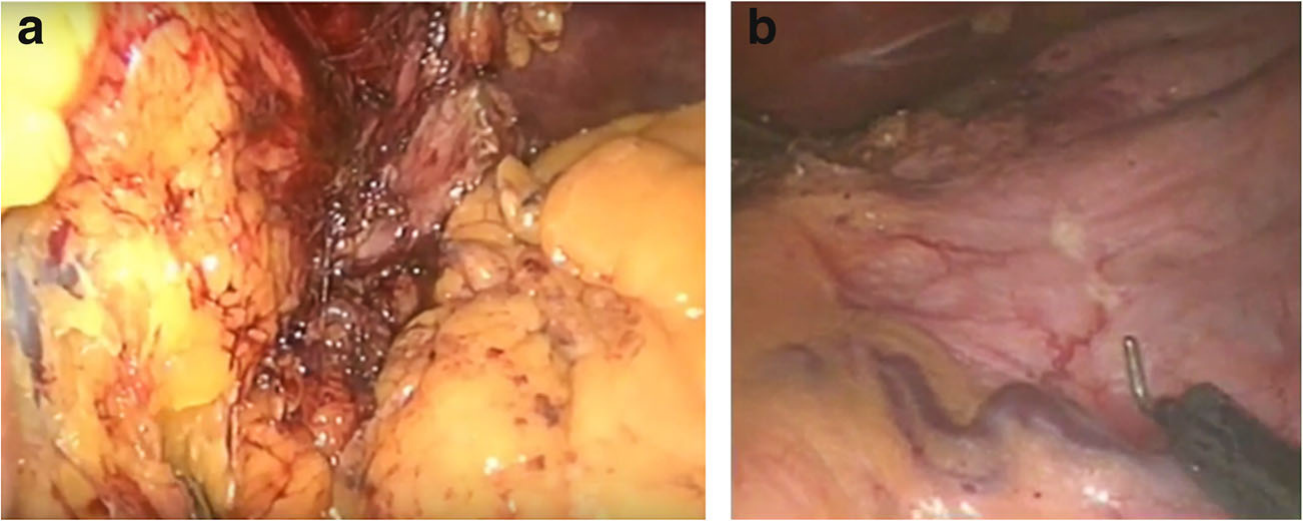
**a** The extent of the posterior dissection is shown, where the left gastric vascular bundle is exposed and all the membranous attachments cephalic to it are freed up, to the left esophageal crus, to completely free the fundus of the stomach. **b** Marking of the resection line with low-powered Hook monopolar electrosurgery. The marking starts at 0.5 cm lateral to the angle of His, in a semi-stretched stomach, parallel to the lesser curvature, down to the antrum at a 3–4-cm distance from the pyloric ring

**Fig. 2:**
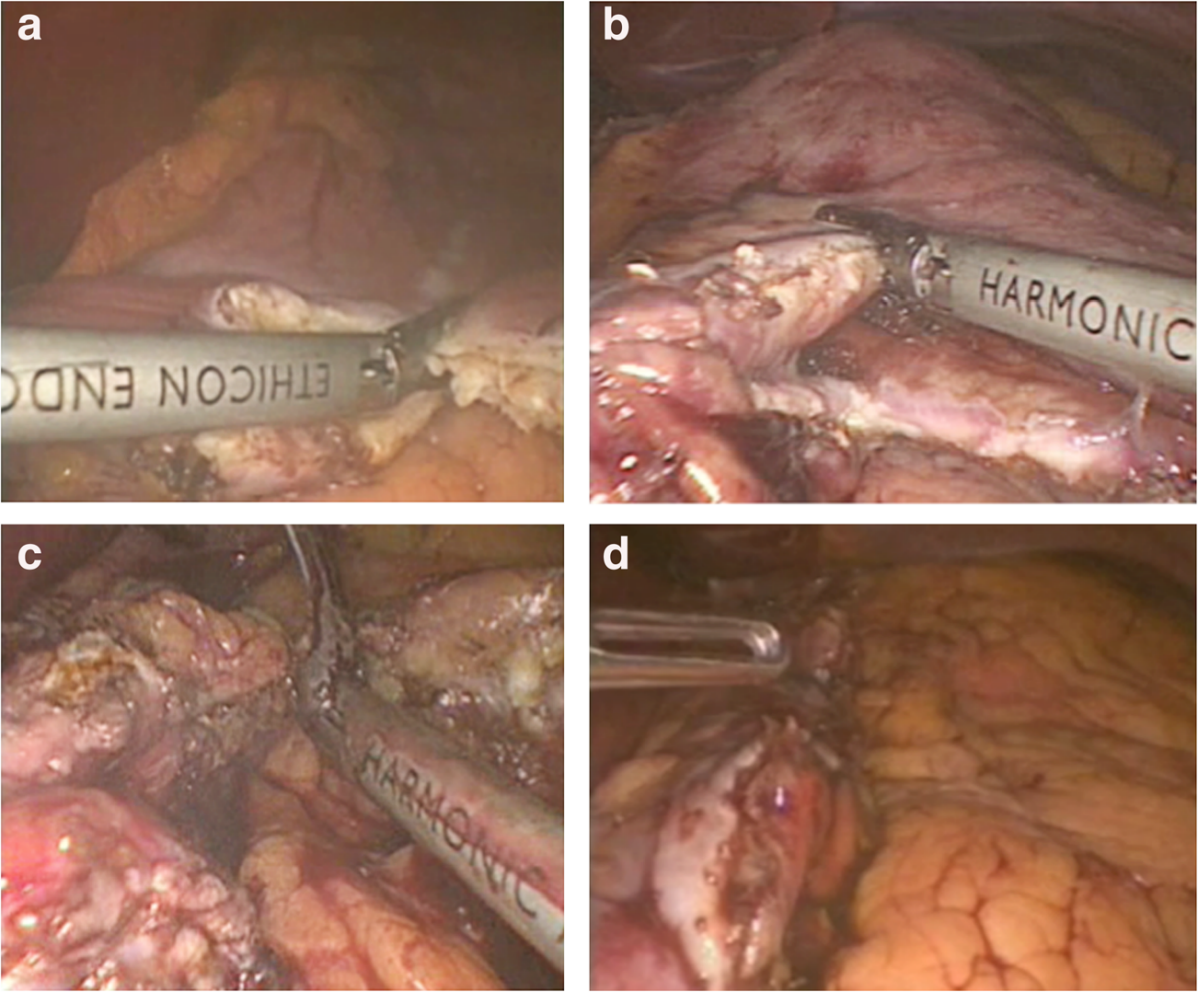
**a** Harmonic coming from the left-hand side to transect the stomach 3–4 cm from the pylorus. **b** Harmonic coming from the right hand side at the incisura, to achieve ergonomic performance. However, due to the jaw dimensions, each wall had to be taken down separately. **c** Last cut of the harmonic applied on the semi-stretched stomach, to ensure that there is enough tissue to suture, without plunging it into the junction, to avoid obstruction. **d** Overview on the pouch prior to start of first layer

**Fig. 3:**
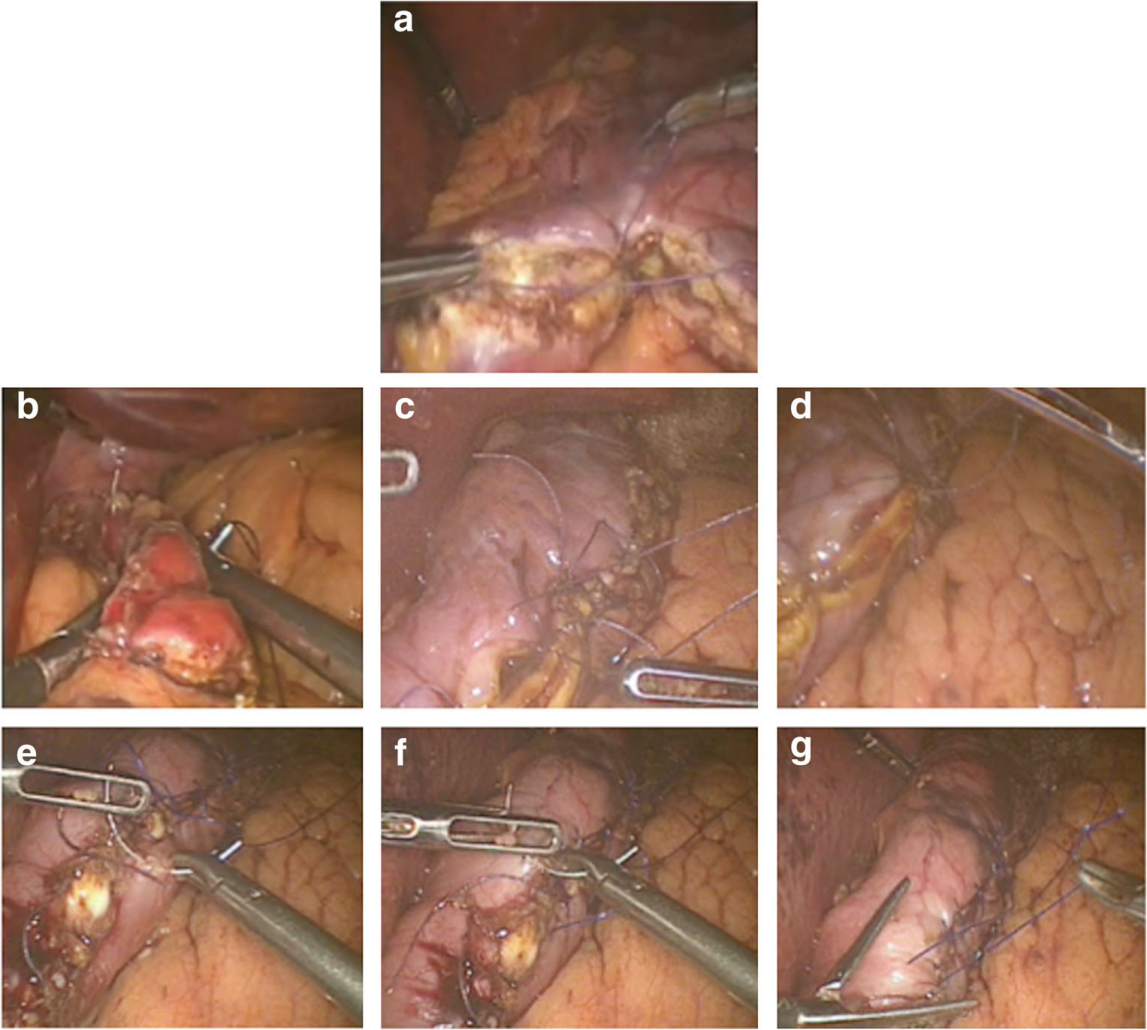
**a** The first stay suture made 4–5 cm after cutting through the stomach with the harmonic scalpel. **b** Start of the full thickness first layer just above the cut angle at the angle of His. **c** and **d** Stationed (tie between the running full thickness and any one of the stay). **e** and **f** Second continuous invaginating seromuscular layer of sutures being taken

The modification was conceived in 2011 by MB, who also performed the subsequent procedures. After proper dissection (Fig. 1a), we marked the resection line (Fig. 1b), after insertion of a 38-Fr bougie. Then, the harmonic scalpel was used from the left-hand port (Fig. 2a) to transect the stomach 3–4 cm from the pylorus. At the incisura, the harmonic was used from the right surgical port (Fig. 2b) to achieve ergonomic performance. A step suture (Fig. 3a) was taken every 4–6 strokes of the harmonic to act as stations for the continuous suture and to allow for better resection throughout. After completion of the resection (Fig. 2d), a full thickness layer—stationed at the interrupted sutures—was taken (Fig. 3b–d). Subsequently, a second continuous invaginating seromuscular layer was taken (Fig. 3e–g).

**Fig. 4:**
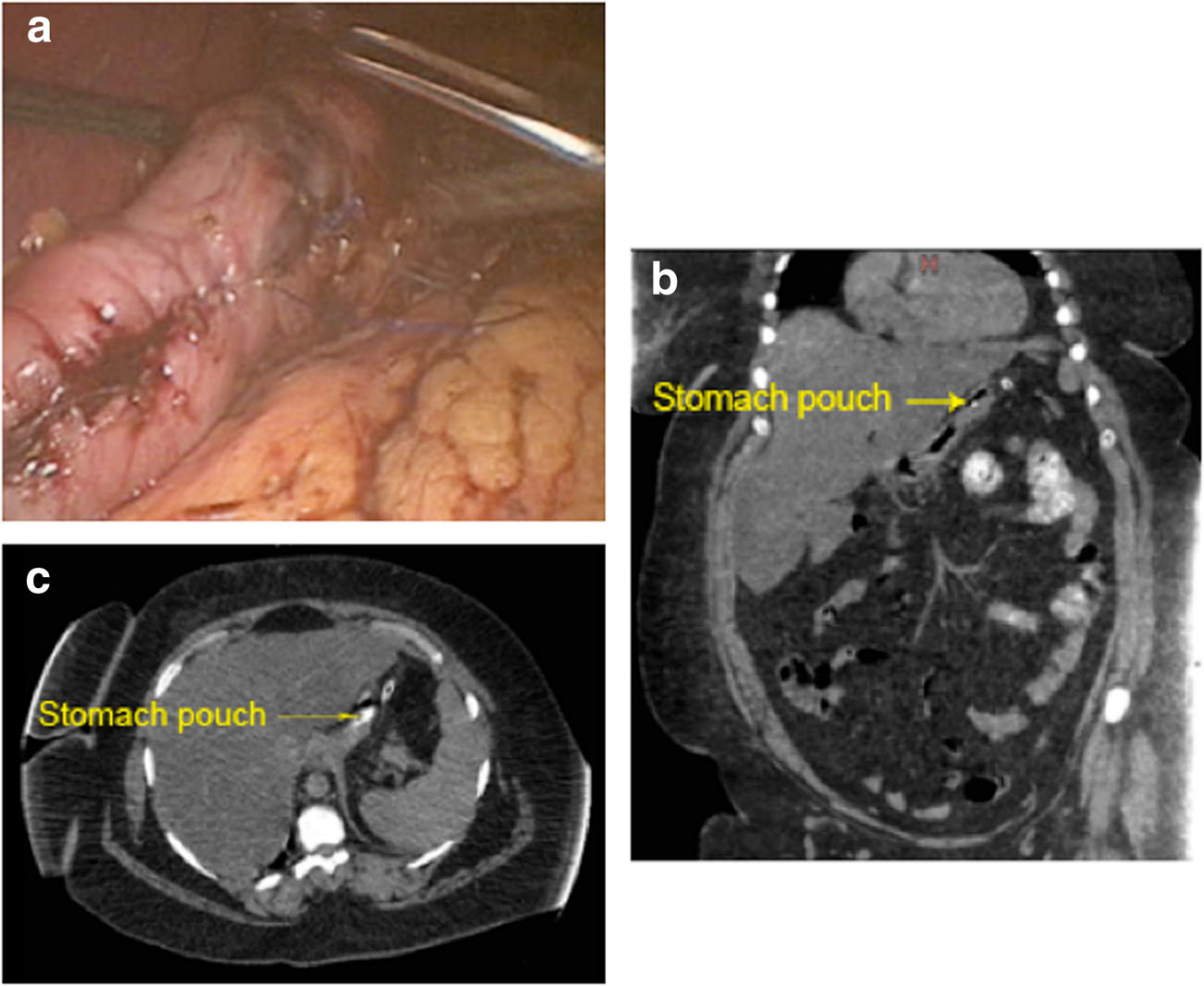
**a** Global view at the end of the procedure. Post-operative CT scan with oral contrast (**b** axial and **c** coronal views)

A methylene blue leak test was performed at the end of the procedure (Fig. 4c) to ensure the tightness of sutures.

Patients were positioned in a steep anti-Trendelenburg position with the pneumoperitoneum established through a 12-mm trocar, inserted a handbreadth (13–14 cm) beneath the xiphoid process, and minimally deviated to the left of the midline. A second 12-mm optical trocar was inserted two fingers breadth beneath the costal margin just at the left midclavicular line. Three 5-mm trocars were inserted: one subxiphoid for liver retraction and manipulation of the gastric fundus when needed, another one at the left midaxillary line for the assistant, and the third one in the right pararectal line, two fingers breadth below the costal margin. The last one may transfix the falciform ligament if found broad and long.

Dissection was pursued using ultrasonic dissector (Harmonic Ace; Ethicon Endo-Surgery, USA) through accessing the lesser sac; then, the whole greater curvature of the stomach was dissected. Afterward, complete liberation of the posterior gastric attachments except for the single left gastric vessel bundle was performed. All the remaining fat, peritoneal bands, and posterior fundic vessels were freed from their gastric attachment (Fig. 1a). Complete exposure of the left crus is gained, and mobilization of the angle of His is completed through dissection of the phrenogastric membrane from the left side until the gastroesophageal junction is mobilized.

The pouch was designed drawing a line 0.5 cm lateral to the angle of His, running parallel to the lesser curvature to 3–4 cm from the pylorus—depending on how far the pylorus is shifted to the right side. Traditionally, we used to staple the pouch just at the angle of His running through a parallel line to the lesser curvature. However, in our case series, the lateral extra 0.5 cm was allowed, to give space for the second layer of sutures. The pouch size was calibrated over a 38-Fr size calibrating bougie. Initial marking of the resection line was done with a low-powered Hook monopolar electrosurgery (Fig. 1b). This line marked the second suture line and was marked under a moderate stretch of the stomach to obtain a geometrically homogeneous tube without ambiguity—which we believe is one of the most common causes of poor outcome following surgery. Furthermore, the distance between the full thickness and the imbricating suture layers was designed to prevent excess tissue invagination which would otherwise produce internal inhomogeneity in the tube.

The resection was started 3–4 cm from the pylorus through the paramedian trocar with ultrasonic dissector (Harmonic Ace; Ethicon Endo-Surgery, USA) and was performed with a 38-Fr calibrating tube inside the pouch (Fig. 2a, b). The resection was continued until we reached the angle of His through the same port (Fig. 2c). During resection, traction was applied at the greater gastric curvature and was slightly pulled toward the anterior abdominal wall to remove the relatively larger surface of the posterior wall of the gastric fundus.

A few permanent, full thickness, sutures, using Vicryl 3/0 mounted on a 26–30-mm round needle, were taken 2–3 mm lateral to the resection line (Fig. 3a), every 4–6 strokes of the harmonic, to act as stations for the continuous suture, as well as to better control the resection and design the pouch throughout (Ethicon Sutures, Cincinnati, OH, USA).

Full thickness sutures—stationed at the interrupted sutures—were taken full thickness at 2–3 mm lateral to the resection line, in a continuous manner (Fig. 3b–d). The sutures covered the entire resection line, using Vicryl 3/0, 26–30 mm mounted on a round needle (Ethicon Sutures, Cincinnati, OH, USA). Invaginating sutures were taken into the superficial seromuscular layer, 2–3 mm lateral to the first layer, in a continuous manner. (Fig. 3e–g) The sutures covered the entire resection line, using polypropylene 3/0, 26–30 mm round needle (Ethicon Sutures, Cincinnati, OH, USA).

A leak test with diluted methylene blue was performed, and a tube drain was left adjacent to the gastric pouch [8]. Since early leakage usually presents with subtle symptoms [7], a post-operative computed tomography (CT) with oral contrast was routinely adopted in this series (at 30–48 h post-op), to verify the absence of leak and demonstrate the appropriate size of the pouch (Fig. 4b, c).

## Results

This case series looked at three middle-aged women (median age 42 years; range 39–51 years) suffering from super obesity (median body mass index (BMI) 50 kg/m^2^; range 49–53 kg/m^2^) (Table 1). Patients B and C had similar co-morbidity profiles, except for patient B having had previous abdominal surgery, and patient C had hypertension (HTN). Patient A, however, had obstructive sleep apnea (OSA), hypertension, recurrent incisional hernia, and previous abdominal surgery with a mesh. None of the patients suffered from diabetes or hiatus hernia (Table 1). The median operative time was 132 min (range 120–195 min) (Table 1). Post-operatively, all patients stayed for 2 days in the hospital and there was no leakage on the routine post-operative (30–48 h) CT (Table 1). Median excess weight loss (EWL) at 6 months was 39% (range 32–43%) and at 1 year 57.7% (range 50.4–63%). A delayed leakage (7–10 days post-operatively)—which could be attributed to heat-related sloughing of the gastric wall—was excluded on a clinical basis during the initial follow-up period that was extended to 30-days post-operatively.

**Table 1:** Demographics and peri-operative characteristics of three patients who underwent stapleless laparoscopic sleeve gastrectomy

	Patient A	Patient B	Patient C
Characteristic			
Age (years)	39	51	42
Body mass index (BMI)	52.67	50.31	49.31
Gender (f = 0; m = 1)	0	0	0
Pre-op risk factor			
Diabetes (no = 0; yes = 1)	0	0	0
Obstructive sleep apnea (no = 0; yes = 1)	1	0	0
Hypertension (no = 0; yes = 1)	1	0	1
Reflux (no = 0; yes = 1)	0	0	0
Previous abdominal surgery (no = 0; yes = 1)	1	1	0
Current hernia (no = 0; yes = 1)	1	0	0
Hiatal hernia (no = 0; yes = 1)	0	0	0
Peri-op technical consideration			
Instrument	Harmonic	Harmonic	Harmonic
Number of layers	2	2	2
Fashion	Stationed/continuous	Stationed/continuous	Stationed/continuous
Post-op CT (no = 0; yes = 1)	1	1	1
Main outcomes measured			
Operative time (minutes)	195	120	132
Leak (no = 0; yes = 1)	0	0	0
Hospital stay (days)	2	2	2
BMI 6 months (EWL%)	36.39 (51%)	37.56 (36%)	34.33 (48%)
BMI 1 year (EWL%)	34.2 (71%)	29 (53%)	33.9* (59%)

*Weight loss information is available for this patient at 10 months only

All patients had some form of post-operative food intolerance. The commonest symptom among all patients was post-operative anorexia. All patients described nausea secondary to food odor; additionally, all patients experienced vomitus at least once post-operatively.

### Discussion

Stapleless LSG is a potentially safe and efficient alternative to the standard stapled LSG. Stapleless LSG has not been widely discussed in literature. However, a recent comment on stapleless laparoscopic Roux-en-Y gastric bypass (LRYGBP), exemplifies some of the hypothetical critiques of stapleless LSG, stating that “*the potential advantage of avoiding leaks and fistulas may not be consistent overall*,” describing it as “*cumbersome and time-consuming*,” and finally that “*the reductions in cost may not be worth it if complication rates increase*” [9]. Given the dearth of literature looking specifically at stapleless LSG [10, 11], we will look at LSG with oversewing as a proxy. We will place the hypothetical critiques in the context of the broader literature and address them one by one.

Some have doubted whether SLR by oversewing does carry any advantages in leak rates [12], bleeding rates [13], or both [14], compared to LSG alone. They claim that some studies do not show statistically significant results, or that oversewing might increase bleeding rates for example. Firstly, this is contested by studies which point to a clear reduction in leak rates [15], bleeding rates [16], and both [17, 18]. However, more importantly, the problem with many of the studies that cast doubt over SLR by oversewing is that they lack statistical power [18]. This is in part because leak and bleeding rates are currently quite low to start with, especially once the learning curve has been overcome. Additionally, Chen et al. remarked that a large sample size (around 9346 procedures) would be needed to detect relatively significant differences in leak rate [19]. None of the studies reviewed critiquing oversewing met this threshold. Whereas, the one meta-analysis that did meet that sample size threshold (Shikora and Mahoney 2015), showed a statistically significant link between oversewing and reduced leak and bleeding rates [18].

Some might suggest that a stapleless method has increased risk of leaks and hemorrhage as the surgeon needs to open the stomach and then close it with sutures. This was alluded to by Póvoas and Vilas-Bôas (2006) when describing the complication that arose following their attempt at Stapleless RYGBP [9]. Again, no literature could be found comparing a hand-sewn versus a stapled technique for closure of the stomach in LSG. Therefore, we used evidence surrounding gastrojejunostomy anastomosis creation as a proxy for this. Most of the studies reviewed compared hand-sewn anastomosis (HSA) with circular-stapled anastomosis (CSA) and linear-stapled anastomosis (LSA). CSA was ignored as these are not used in LSG.

Initial studies were inconclusive. Some are suggesting that HSA has a higher stenosis rate [20], whereas others showed lower rates [21]. Some these studies employed three different procedures early in the learning curve, which may have confounded the results. More recent evidence has demonstrated no increased risk from hand-sewn versus linear-stapled anastomosis with regards to leaks, bleeding, or stenosis [20, 22]. Extrapolating from this, the evidence seems to suggest that hand-sewn gastric closure would be safe, though studies comparing this with LSA directly will be required.

Oversewing in SLR does increase the operative time when compared with LSG alone [12]. Though this is trivially true, in so far as doing something will always take longer than not doing something, it is also true that oversewing does extend operative time, even when compared to other reinforcement methods [23]. This was also true of our series, where the median operative time was 132 min (range 120–195 min), with patient A, the most obese and co-morbid, taking the longest, 195 min (Table 1). However, historically, surgery had often opted for more time-consuming techniques when benefits outweighed this [24]. Moreover, it is unclear how big a problem this is in practice as one group reported comparable operative times with and without oversewing [15, 18]. Finally, this factor can be easily reduced with experience, especially in high-volume centers [18]. The same meta-analysis also showed that buttressing produced slightly better outcomes than oversewing. Unfortunately, these slight benefits need to be weighed against costs. Among different techniques for reinforcement, oversewing is seen to be the most affordable one [25]. Some have reported that for large institutional hospitals, buttressing materials (e.g., absorbable polymer membranes, bovine pericardial strips, or fibrin sealants) are too expensive for use on a permanent basis; therefore, suturing is the best option regarding costs and benefits [18]. This is particularly the case in countries with limited income per capita, as highlighted by Ettinger et al. using Brazil as an example. They explain that because materials are imported incurring taxes and currency exchange rates, the final cost is expensive compared with other methods. They illustrate this with the following example of the cost of materials for stapled RYGBP: the stapler for the laparoscopic RYGBP in Salvador, Brazil, is R$3220 (Brazilian Reals) = US$1340 (US dollars). A laparoscopic cartridge costs R$1260 = US$525. The total cost per operation using one stapler plus seven cartridges is R$12,040 = US$5016 [26].

When these factors are taken together, a hand-sewn technique becomes not only better than LSG alone regarding outcomes but is also likely to be more cost-effective, when compared to LSG with no reinforcement and LSG with buttressing.

Unsurprisingly during the early post-operative period, most of the patients had symptoms related to food intolerance. However, the clinical impression is that this was not different from the standard sleeve gastrectomy [27]. It is, of course, obvious that no reliable statistics could be generated from this small sample size. In future, research could be carried out using validated questionnaires [28] to explore whether stapleless LSG has any effect on food tolerance.

A substantial modification of this method is the addition of invaginating sutures over underrunning continuous sutures. Reinforcement with invaginating sutures has been shown to have reduced the leak rate from around 7 to 0% in one our previous study [8]. The study by Rogula et al. (2015) [25] showed a decrease in leak rate by 70 to 0% in vitro, when comparing imbricating sutures and continuous through-and-through sutures [24].

We propose that a single seromuscular (extra mucosal) layer could be sufficient; however, we have yet to test this hypothesis. The purpose of the two-layer suturing adopted here is to protect against energy leakage from energy induced sloughing of the gastric wall. The lateral thermal spread on the gastric wall from the harmonic is not known to the best of our knowledge. However, one of the advertising proclamations of the harmonic is the minimal lateral spread [29]. It is worth remembering that the thermal lateral spread is theoretically larger than what should be on the stomach, such as porcine carotid arteries [30] and bovine muscle fascia [29]. Thus, given the hemostatic nature of the instrument, we think that the full thickness layer—taken for hemostasis mainly—can be dispensed. This is also an analogy to the bowel anastomosis in which single layer is proved to be as effective as two layers [31].

In our limited case series, we routinely performed CT scan with oral contrast to detect any leak. We acknowledge that the cost of this extra imaging increases the cost of our stapleless technique; however, we felt that it was warranted in our initial series to be skeptical. Although our patients were closely monitored for clinical signs of early leak such as tachycardia, fever, and unequal inspiration [7], we note that these signs are not specific to leakage and could indicate atelectasis, which is recognized as the most common complication in bariatric patients [32]. Dehydration also causes low-grade pyrexia and tachycardia and could have confounded the diagnosis of an early leak. We found our patients were vulnerable to dehydration, as post-operative nausea and vomiting was a frequent event. An additional reason to perform CT scans in our study was to assess the geometry of the pouch.

### Limitations

In this study, we used the harmonic scalpel. We recognize that this may be a limited instrument for this procedure. It has a small jaw size, which cannot grasp both gastric walls (anterior and posterior) together in certain areas. This affects the surgical technique, as it requires considerable attention during the resection to optimally fashion the pouch and prevent discrepancy between the walls. This could affect the geometry of the pouch which could adversely affect outcomes.

In this proof of concept study, there was neither leak nor bleeding from surgery. However, a powered study is required to fully investigate the merits of this procedure.

## Conclusion

Stapleless LSG seems to have the potential to be an affordable alternative to the standard LSG techniques, both regarding financial costs and complications rate. High-powered studies and formal cost analyses will be required looking specifically at the stapleless method versus the standard LSG.
